# Contribution of the dihydropyrimidinase-like proteins family in synaptic physiology and in neurodevelopmental disorders

**DOI:** 10.3389/fnins.2023.1154446

**Published:** 2023-04-17

**Authors:** Florence Desprez, Dévina C. Ung, Patrick Vourc’h, Médéric Jeanne, Frédéric Laumonnier

**Affiliations:** ^1^UMR1253, iBrain, Inserm, University of Tours, Tours, France; ^2^Service de Génétique, Centre Hospitalier Régional Universitaire, Tours, France; ^3^Laboratoire de Biochimie et de Biologie Moléculaire, Centre Hospitalier Régional Universitaire, Tours, France

**Keywords:** dihydropyrimidinase-like proteins, collapsin response mediator proteins, neurodevelopmental disorders (NDDs), human genetics research, neuronal development, synaptic physiopathology, missense variants, animal model

## Abstract

The dihydropyrimidinase-like (DPYSL) proteins, also designated as the collapsin response mediators (CRMP) proteins, constitute a family of five cytosolic phosphoproteins abundantly expressed in the developing nervous system but down-regulated in the adult mouse brain. The DPYSL proteins were initially identified as effectors of semaphorin 3A (Sema3A) signaling and consequently involved in regulation of growth cone collapse in young developing neurons. To date, it has been established that DPYSL proteins mediate signals for numerous intracellular/extracellular pathways and play major roles in variety of cellular process including cell migration, neurite extension, axonal guidance, dendritic spine development and synaptic plasticity through their phosphorylation status. The roles of DPYSL proteins at early stages of brain development have been described in the past years, particularly for DPYSL2 and DPYSL5 proteins. The recent characterization of pathogenic genetic variants in *DPYSL2* and in *DPYSL5* human genes associated with intellectual disability and brain malformations, such as agenesis of the corpus callosum and cerebellar dysplasia, highlighted the pivotal role of these actors in the fundamental processes of brain formation and organization. In this review, we sought to establish a detailed update on the knowledge regarding the functions of *DPYSL* genes and proteins in brain and to highlight their involvement in synaptic processing in later stages of neurodevelopment, as well as their particular contribution in human neurodevelopmental disorders (NDDs), such as autism spectrum disorders (ASD) and intellectual disability (ID).

## 1. Introduction

The Dihydropyrimidinase-like (DPYSL) proteins, also designated as the Collapsin response mediators (CRMP) proteins, constitute a family of five cytosolic phosphoproteins (Quinn et al., 1999), abundantly expressed in the developing nervous system but down-regulated in the adult mouse brain (Minturn et al., 1995; Byk et al., 1996; Wang and Strittmatter, 1996; Fukada et al., 2000; Yuasa-Kawada et al., 2003). The DPYSL proteins were initially identified as effectors of semaphorin 3A (Sema3A) signaling and consequently involved in regulation of growth cone collapse (Goshima et al., 1995).

To date, it has been established that DPYSL proteins mediate signals for numerous intracellular/extracellular pathways and play major roles in variety of cellular process including cell migration (Yamashita et al., 2006), neurite extension (Brot et al., 2010), axonal guidance (Goshima et al., 1995; Arimura et al., 2005; Uchida and Goshima, 2005; Yoshimura et al., 2005), dendritic spine development (Yamashita et al., 2007) and synaptic plasticity (Yamashita et al., 2011) through their phosphorylation status.

The roles of DPYSL at early stages of brain development have been described in the past years, particularly for DPYSL2 and DPYSL5. In this review, we sought to establish a detailed synthesis on the functions of DPYSL genes and proteins in brain and to highlight their involvement in synaptic processing in later stages of neurodevelopment, as well as their contribution in human neurodevelopmental disorders (NDDs), such as autism spectrum disorders (ASD) and intellectual disability (ID). This synthesis will likely provide a new perspective regarding the specific function of *DPYSL* genes and proteins in developing and functioning brain, as well as their respective role in the fine regulation of brain developmental stages.

## 2. The DPYSL proteins family

In mammals, five DPYSL (or CRMP) proteins have been identified and are encoded by their respective coding-genes (*CRMP1 or DPYSL1*, *DPYSL2, DPYSL3, DPYSL4, and DPYSL5*) (Table 1). The predicted secondary structure of human DPYSL proteins family is well described and allowed to determine that DPYSL1-4 display around 75% sequence homology with each other, whereas DPYSL5 is more distant phylogenetically with only 50% of sequence homology (Schmidt and Strittmatter, 2007; Tang et al., 2015). Alignment and comparison of amino acid sequence of mouse DPYSL5 with other DPYSL proteins show that the conservation level is lower at the C-terminal region (Fukada et al., 2000; Tang et al., 2015). The DPYSL associate generally in homo-tetramer or hetero-tetramer complexes with single or multiple isoforms.

**Table 1:** List of known human *DPYSL/CRMP* genes and corresponding proteins.

Human gene (HGNC)	Locus	Transcript RefSeq	Human protein	Alias for protein name	Protein RefSeq
*CRMP1*	4p16.2	NM_001014809.3	DPYSL1	CRMP1	NP_001014809.1
*DPYSL2*	8p21.2	NM_001197293.3	DPYSL2	CRMP2	NP_001184222.1
*DPYSL3*	5q32	NM_001197294.2	DPYSL3	CRMP4	NP_001184223.1
*DPYSL4*	10q26.3	NM_006426.3	DPYSL4	CRMP3	NP_006417.2
*DPYSL5*	2p23.3	NM_001253723.2	DPYSL5	CRMP5	NP_001240652.1

The official name of the human genes is indicated according to the Hugo Gene Nomenclature Committee (HGNC) nomenclature. The chromosomal locus of each gene corresponds to the Genome Reference Consortium Human Build 38 patch release 14 (GRCh38.p14). The reference sequence (RefSeq) for genes and protein was extracted from the National Center for Biotechnology Information (NCBI) database.

## 3. Physiological pathways involving DPYSL proteins

The DPYSL proteins participate in several major physiological pathways, from cellular migration, neurite growth and guidance to synapse maturation, particularly through their C-terminal domain, which includes the last 50 amino-acids, and which is the target of numerous post-translational modifications sites that regulate the interaction between DPYSL and various types of proteins, including receptors, ion channels, cytoskeletal and motor proteins.

As an example, numerous kinases such as glycogen kinase 3β (GSK3β), cyclin-dependent kinase 5 (Cdk5), dual specificity tyrosine phosphorylation-regulated kinase 2 (DYRK2) and Rho-associated kinase 2 (ROCK2) target the C-terminal regions of DPYSL proteins (Uchida and Goshima, 2005; Yoshimura et al., 2005; Cole et al., 2006; Arimura and Kaibuchi, 2007; Uchida et al., 2009; Yamashita and Goshima, 2012; Nakamura et al., 2014). Despite differences in the C-terminal part of DPYSL5 compared to other DPYSL proteins, it is noted that many consensus sequences for DPYSL phosphorylation sites are retrieved for DPYSL5 (Fukada et al., 2000).

The C-terminal domain is extensively conserved among DPYSL isoforms and across species, and is sufficient to associate with assembled microtubules *in vivo* (Soutar et al., 2009). Precisely, phosphorylation/dephosphorylation status of DPYSL proteins is essential to control their spatiotemporal functions, by modulating their binding to cytoskeleton and signaling proteins (Yamashita and Goshima, 2012).

Thus, DPYSL proteins can coordinate cytoskeleton dynamic regulating filopodia formation, axonal guidance, neurite outgrowth and establishment of neuronal polarity by interacting with tubulin and actin in brain (Fukata et al., 2002; Arimura et al., 2005; Hotta et al., 2005; Rosslenbroich et al., 2005; Brot et al., 2010; Higurashi et al., 2012; Ji et al., 2014; Khazaei et al., 2014; Tan et al., 2015; Gong et al., 2016; Yu-Kemp and Brieher, 2016). Non-phosphorylated DPYSL2 promotes axonal elongation and branching by binding to tubulin heterodimer (Schmidt and Strittmatter, 2007) whereas its phosphorylation by GSK3β, ROCK2 and Cdk5 lowers binding affinity of DPYSL2 to tubulin leading to growth cone collapse and arrest of axonal outgrowth (Fukata et al., 2002; Schmidt and Strittmatter, 2007; Khanna et al., 2012). The binding of DPYSL1-3 to tubulin allows polymerization and stabilization of microtubules (Fukata et al., 2002; Lin et al., 2011; Khazaei et al., 2014) while the tubulin-DPYSL4 or tubulin-DPYSL5 complex interaction causes inhibition of microtubule polymerization (Aylsworth et al., 2009; Brot et al., 2010).

In addition to phosphorylation, the functions of DPYSL proteins are also regulated by other post-translational modifications including acylation, SUMOylation and O-GlcNAcylation (Leney et al., 2017; Myllykoski et al., 2017; Chew and Khanna, 2018). For instance, DPYSL2 phosphorylation at Serine 522 by Cdk5 promotes association between DPYSL2 and cytoplasmic loops of CaV2.2 (Brittain et al., 2012; Chew and Khanna, 2018), leading to an increase of Ca^2+^ influx through the Cav2.2 channel and the release of neurotransmitters (Brittain et al., 2009, 2012). Similarly, SUMOylation of DPYSL2 alters calcium influx (Ju et al., 2013) and increases cell surface expression of NaV1.7 channel (Dustrude et al., 2016). Dephosphorylation of DPYSL2 at Thr514 and deSUMOylation at Lys374 sites promote the formation and maturation of dendritic spines, however, no interference is found between these two post-translational modifications in the regulation of dendritic spine morphology (Zhang et al., 2018).

## 4. Neuronal expression of DPYSL genes and proteins

Based on *in situ* hybridization (Wang and Strittmatter, 1996) and immunostaining analyses (Bretin et al., 2005), the DPYSL proteins are detected at a higher level in post-mitotic neural cells during the embryonic stage than in adult mouse brain stage (Wang and Strittmatter, 1997; Ricard et al., 2001). The mRNA expression level of *Dpysl* genes is intense during the neonatal period (Embryonic day 18 – Postnatal day 5) in the central nervous system of mice (Charrier et al., 2003). At Postnatal day 1, all DPYSL except DPYSL4 are strongly expressed in cortex and hippocampus (Wang and Strittmatter, 1996; Ricard et al., 2001) essential for social communication and cognitive functions. In addition, a peak of the expression level of DPYSL proteins is observed during the first postnatal week corresponding to a period of neuronal maturation and synaptogenesis (Byk et al., 1996; Wang and Strittmatter, 1996; Bretin et al., 2005; Schmidt and Strittmatter, 2007).

**Figure 1:**
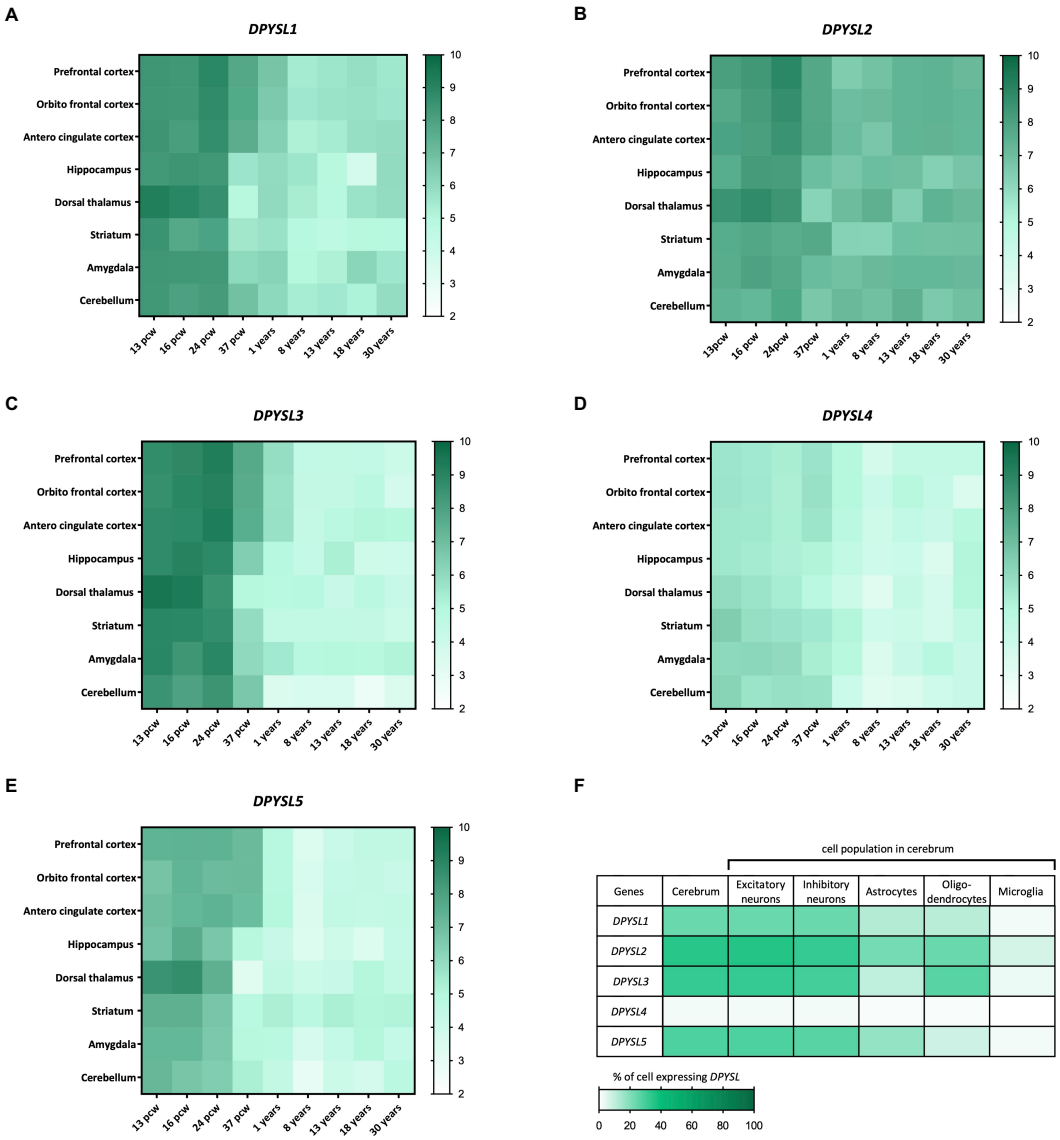
Dynamic expression profile of *DPYSL* genes in the human brain. **(A–E)** Spatio-temporal profile of expression of *DPYSL* genes. The database used to perform the gene expression heatmap is BrainSpan. The level expression is expressed in RPKM (reads per kilobase of transcript per million reads mapped) and these data are obtained from RNA sequencing and exon microarray. Pcw, post-conceptual week. **(F)** *DPYSL* expression in different cell populations of the humain cerebrum. Data are from BBI allen single cell atlases. The single-cell atlases is realized from human fetal samples (72–129 days post-conceptual age).

The BrainSpan transcriptome of the developing human brain shows a similar kinetics of *DPYSL* genes expression level, indicating their preponderant role in prenatal and perinatal periods when neurogenesis, dendritic development and synaptogenesis stages occur (Figures 1A–E) (Sunkin et al., 2013). Interestingly, DPYSL2 displays similar expression levels from prenatal to adult stages, suggesting that it may also have a role in later stages of development such as in myelination or synaptic pruning (Figures 1A–E). Indeed, DPYSL2 mediates Semaphorin 3F dependent synapse pruning (Ziak et al., 2020).

A transcriptomic study of human fetal brain development (BBI Allen single cell atlases) using single-cell RNA sequencing indicated that around 25–30% of cells display *DPYSL expression* in cerebrum, except *DPYSL4* which is very weakly expressed (only 2% of cells) (Cao et al., 2020). *DPYSL* mRNAs are mainly found in neurons (excitatory/inhibitory) but also in glial cells (Figure 1F), which suggest a contribution in neuronal degeneration/regeneration, as well as in inflammatory pathways in the context of neurological diseases inflammation and neurodegeneration pathways (Nagai et al., 2017).

## 5. Antagonistic/synergistic roles of DPYSL proteins

Each DPYSL protein displays a distinct subcellular neuronal localization both in time and space demonstrating their divergent functions during development (Goshima et al., 1995; Minturn et al., 1995; Byk et al., 1996; Wang and Strittmatter, 1996; Byk et al., 1998; Kamata et al., 1998; Bretin et al., 2005).

In primary hippocampal mouse neurons, during the axonogenesis, DPYSL2 is specifically enriched in neurite which is the future axon while DPYSL5 is strongly retrieved in dendrites maintaining dendrites at a quiescent state (Brot et al., 2010, 2014). At Days *in vitro* (DIV) 4–5, a switch is observed. DPYSL5 is detected at a very low level in dendrites and DPYSL2 level remains constant in same area allowing dendritic outgrowth (Brot et al., 2010). The transient DPYSL5 expression in different neuronal compartments regulates the establishment of neuronal polarity (Bretin et al., 2005; Brot et al., 2010, 2014). DPYSL5 forms a ternary complex with tubulin and microtubule associated protein 2 (MAP2) and inhibits the neurites outgrowth by reducing DPYSL2-tubulin interaction complex (Brot et al., 2010; Brot, 2014). It is not yet excluded that the inhibition of DPYSL2 activity by DPYSL5 may occur through their hetero-oligomerization as DPYSL2 and DPYSL5 have a very similar structure and form hetero-tetramer *in vivo* (Fukada et al., 2000; Brot et al., 2010; Petratos and Lee, 2013; Ponnusamy and Lohkamp, 2013).

Despite that DPYSL5 does not inhibit axonal growth (Brot et al., 2010), its deficiency results in an increase on DPYSL2-induced axon elongation and on multiple axon formation (Inagaki et al., 2001). These results suggest that *in vivo* DPYSL5 also modulates DPYSL2 activity on axonal growth and formation.

Although the biological functions associated with each homo- or hetero-tetramer of DPYSL proteins are still poorly known, it has been demonstrated that DPYSL2 and DPYSL3 are complexing and work together to regulate growth cone development and axonal elongation *in vivo* (Tan et al., 2015). For instance, overexpression of DPYSL2 and DPYSL3 in hippocampus stimulate axonal growth and this effect is abolished when DPYSL2 is co-transfected with the truncated construct DPYSL3ΔC471 (unable to bind actin) or when DPYSL3 is co-transfected with DPYSL2ΔC322 (unable to bind tubulin). These findings suggest that DPYSL2/DPYSL3 hetero-tetramer complex creates a link between microtubules and actin, aiming to coordinate cytoskeleton dynamics, and axonal development regulation in hippocampal neurons (Tan et al., 2015). These findings illustrate the various actions of DPYSL proteins and highlight their ability to form homo- or hetero-tetramer complex, in order to modulate and regulate the function of other DPYSL proteins during neural network formation.

DPYSL proteins appear to play antagonistic but also complementary roles during neurodevelopment (Byk et al., 1998; Yuasa-Kawada et al., 2003; Brot, 2014; Makihara et al., 2016). *In vivo* studies demonstrate that DPYSL1 and DPYSL2 have synergistic but distinct roles in mediating Sema3A signaling in order to regulate dendritic development and spine maturation (Hamajima et al., 1996; Sasaki et al., 2002; Morita et al., 2006; Yamashita et al., 2007, 2012).

In fact, abnormalities were observed in dendritic patterning (branching and length dendritic) of cortical (layer V) neurons from distinctly *Dpysl1^−/−^* and *Sema3A^−/−^* mouse model, compared to their littermate neurons. These defects in dendritic morphology are not retrieved in KO *Dpysl2^−/−^* and double-heterozygous KO *Dpysl1^+/–^Dpysl2^+/−^* mouse models. Moreover, the level of DPYSL1 increases in *Dpysl2^−/−^* compared to wild-type cortical brain lysates, highlighting a DPYSL1 compensatory mechanism for DPYSL2 deficiency (Diss et al., 2014; Makihara et al., 2016). A proteomic analysis in cortex of *Dpysl2^ki/ki^* mice (where serine 522 is mutated to alanine preventing its potential phosphorylation) demonstrated an increase of DPYSL3, DPYSL4 and DPYSL5 (Nakamura et al., 2018) as well as in *Dpysl2^−/−^* (Nakamura et al., 2016), thereby suggesting that the phosphorylation or loss of functions of DPYSL2 have an impact on other DPYSL proteins.

A study from Yamashita and colleagues showed that both DPYSL1 and DPYSL2 are required for regulating dendritic branch trajectory in cerebral cortical neurons reinforcing their synergistic role in dendritic organization (Yamashita et al., 2012). In addition, DPYSL1-4 may have a redundant role in dendritic growth and maturation in neurons (Quach et al., 2008; Khazaei et al., 2014; Cha et al., 2016; Makihara et al., 2016; Takaya et al., 2017; Kawashima et al., 2021).

## 6. Synaptic functions of DPYSL proteins

### 6.1. Role in the formation and maturation of dendritic spines

When maturation of neurons and synaptic connections is strongly active (around first postnatal week in rodents), DPYSL expression is the highest (Charrier et al., 2003). All five DPYSL proteins are expressed in synaptosomes from rat brain at neonatal postnatal day 1 (P1) (Charrier et al., 2006; Brittain et al., 2009; Yamashita et al., 2012) and are postsynaptic density (PSD) proteins (Collins et al., 2006; Laumonnier et al., 2007), suggesting a role in synaptogenesis and neurotransmission. Dendrites are the first site of synapse formation (Purves and Hume, 1981) and synaptogenesis represents an essential process for the establishment of cognitive and communication function as well as for learning and memory (Elston, 2000).

Studies on genetic deletion of *Dpysl* members in mice establish a direct link between loss of DPYSL and impairment of dendritic patterning and spine development (Table 2; Charrier et al., 2006; Quach et al., 2008; Yamashita et al., 2012). The synaptic density is reduced in *Dpysl1^−/−^*, *Dpysl2^−/−^* mutant mice (Yamashita et al., 2007; Makihara et al., 2016).

**Table 2:** Neuronal and behavioral phenotypes observed in mouse models invalidated for the *Dpysl/Crmp* genes.

Mouse genotype	Neuroanatomical and cellular phenotype	Molecular defect	Electrophysiology	Behavioral phenotype	References
*Dpysl1^−/−^ or Crmp1^−/−^*	- Abnormal dendritic development of CA1 pyramidal neurons - Reduction of synapse density in CA1 hippocampus - Reduced number of mature dendritic spines in cortical neurons	- Decreased PDS95 and GAP-43 protein levels in CA1 hippocampus	- Decreased LTP in CA1 hippocampus	- Hyperactivity, impaired emotional behavior - Decreased pre-pulse inhibition - Impaired learning and memory	Su et al. (2007) and Yamashita et al. (2007, 2013)
*Dpysl2^−/−^ or Crmp2^−/−^*	- Altered dendritic morphology (number spine and dendritic branching) of CA1 pyramidal neurons and cortical neurons (layer V) - Altered dendritic spine pruning in dentate gyrus - Abnormal axon pruning arising from hippocampus and visual cortex - Dysgenesis of corpus callosum	- Abnormal NMDA receptor composition - Decreased level of synaptic proteins NSF, PRKACB, GNAI1, GRIA2, SNAP25 - Increased level of synaptic proteins SHANK3, SHANK2, GRIA1	- Reduced LTP induction in hippocampus	- Decreased anxiety - Hyperactivity - Impaired social behavior, learning and memory - Defects in locomotor activity	Nakamura et al. (2016), Zhang et al. (2018), and Ziak et al. (2020)
*Dpysl3^−/−^ or Crmp4^−/−^*	- Increased dendritic total length and branching in primary hippocampal neurons - Defective infrapyramidal bundle of mossy fibers of the dentate gyrus (DG) pruning in the hippocampus	- Altered mRNA expression levels of genes related to neurotransmission and cell adhesion in hippocampus, cortex and olfactory bulb		*-* Decreased social interaction - Alterations of sensory responses (temperature and olfactory)	Niisato et al. (2012), Tsutiya et al. (2016), Takaya et al., 2017, and Tsutiya et al. (2017)
*Dpysl4^−/−^ or Crmp3^−/−^*	- Abnormal dendrite and spine morphogenesis in hippocampus - Defect in infrapyramidal bundle of mossy fibers pruning in hippocampus		- Impairment of LTP induction in CA1 hippocampus		Quach et al. (2018)
*Dpysl5^−/−^ or Crmp5^−/−^*	- Aberrant Purkinje cell morphology in cerebellum		- Impaired LTD induction between parallel fibers and Purkinje cells	- Abnormal limb-clasping reflexes	Yamashita et al. (2011)

**Figure 2:**
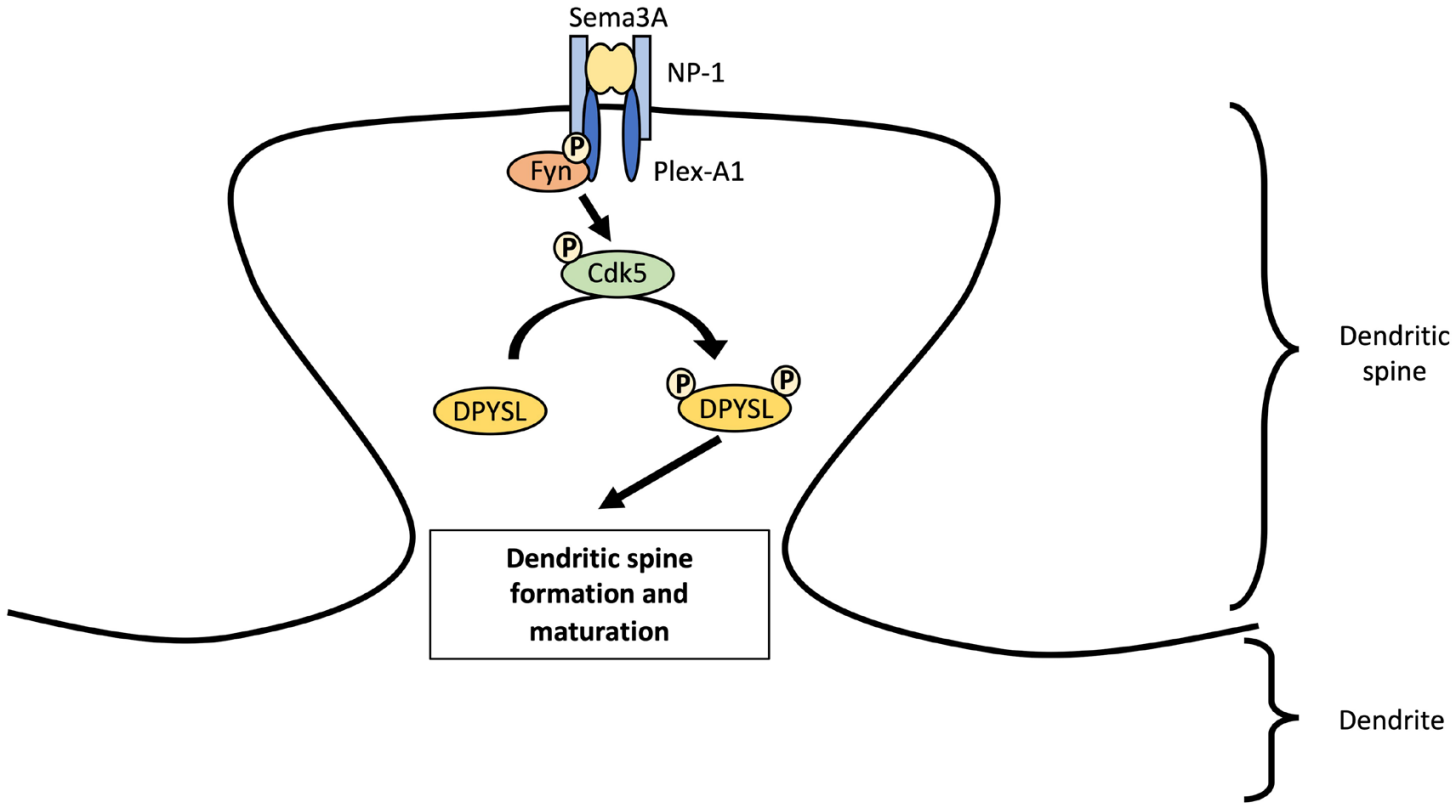
Regulation of dendritic spine maturation in cortical neurons by DPYSL proteins through Sema3A-cdk5 signaling pathway. We propose a model involving the Sema3A-DPYSL pathway in the formation and maturation of dendritic spines. Fyn phosphorylates semaphorin receptor (Plexin A2) and facilitates PlexinA2 interaction to Sema3A. Fyn also promotes the phosphorylation of kinases like cyclin dependent kinase-5 (Cdk5) which in turn phosphorylates DPYSL proteins. We suggest that DPYSL proteins will thus play a role in the genesis and maturation of dendritic spines. AS an example, DPYSL1 can regulate spine development through Sema3A–Cdk5 signaling. Sema3A binds a receptor complex of the transmembrane proteins Neuropilin-1 (NP-1) and Plexin A1 (Plex-A1). After interacting with plexin A1, Fyn stimulates kinase activity of Cdk5 via Tyr15 phosphorylation of Tyr15. Cdk5 Tyr15 phosphorylation will then phosphorylates DPYSL1 tetramer at Thr509 and Ser522 locations. Sema3A through phosphorylation of DPYSL1 by Cdk5 will increase the density of PSD-95 and synapsin I clusters at dendrites and thus, promote formation of mature spines in cultured cortical primary neurons.

The SEMA3A protein is essential for induction of mature spines formation through the Fyn-Cdk5 cascade in cultured cortical neurons (Sasaki et al., 2002; Li et al., 2004; Cole et al., 2006; Morita et al., 2006; Figure 2). Nevertheless, Sema3A is not able to induce an increase in functional synapses density in cortical neurons from *Dpysl1^−/−^* and *Cdk5^−/−^* mice (Yamashita et al., 2007). Several studies revealed the importance of CDK5 phosphorylation of DPYSL1 at Thr509 and Ser522 sites and of DPYSL2 at Ser522 site for SEMA3A-induced spine development and maturation (Yamashita et al., 2007; Jin et al., 2016; Makihara et al., 2016). Conversely, DPYSL2 dephosphorylated forms increase the number of dendritic spines and the amplitude of miniature excitatory postsynaptic currents (mEPSCs) (Zhang et al., 2018). This suggest that dephosphorylated forms of DPYSL2 promotes polymerization of tubulin (Fukata et al., 2002; Uchida and Goshima, 2005) and thus, spinogenesis. A recent study demonstrated that DPYSL2 is not only a mediator of Sema3A-signaling regulating spine development but also plays a key role in synaptic refinement through Semaphorin 3F (Ziak et al., 2020). Loss of *Dpysl2* causes axonal pruning defects and inadequate elimination of dendritic spines in multiples areas of the brain and in cultures of hippocampal neurons (Table 2). This defect is accompanied by social behavior abnormalities (see section “*DPYSL* genes and neurodevelopmental disorders”).

DPYSL3 is also critical for spine formation and maturation in cultured hippocampal neurons *via* the interaction with actin cytoskeleton by its C-terminal region (Rosslenbroich et al., 2005; Cha et al., 2016). Overexpression of DPYSL3 wild-type or DPYSL3 with actin-binding domain constructs increase frequency of mEPSCs in comparison with control GFP or with form of *DPYSL3ΔC471* (lacking the domain of interaction with actin) transfected neurons. These results indicate that DPYSL3-actin interaction increases number of functional synapses and thus, influences synaptic transmission (Cha et al., 2016). Similarly, DPYSL5 deficiency in cerebellum induces an aberrant Purkinje cell morphology. In *Dpysl5^+/−^* mice, Brain-derived neurotrophic factor (*BDNF*) increased the number of primary dendrites per neurons in the hippocampus while this effect is lost in neurons from complete KO *Dpysl5^−/−^* brains (Table 2). Consequently, they demonstrate that DPYSL5 phosphorylation by TrkB is involved in BDNF–TrkB signaling to regulate dendritic morphology and synaptic plasticity in Purkinje cells (Yamashita et al., 2011).

The phosphorylated/dephosphorylated state of DPYSL proteins seems to be crucial for the regulation of their interaction with cytoskeleton proteins and for the control of dendritic architecture (Arimura et al., 2005; Yamashita and Goshima, 2012; Makihara et al., 2016; Zhang et al., 2018). Several post-translational modifications of DPYSL2 allow modulation of membrane addressing of the CaV2.2 and NaV1.7 ion channels, as well as the formation and maturation of dendricic spines (see section Physiological pathways involving DPYSL proteins). These data highlight the importance of future research on their post-translational modifications and associated signaling pathways to clarify their function in synapse formation and in neurotransmission.

### 6.2. Role in physiology and synaptic plasticity

In addition to synapse formation process, DPYSL proteins interact with presynaptic and postsynaptic machinery and may also have a role in synaptic plasticity. For instance, loss of *Dpysl1-4* in murine models cause dysregulation of genes expression related to excitatory and/or inhibitory synaptic transmission explaining synaptic plasticity dysfunction (Yamashita et al., 2011; Tsutiya et al., 2015; Zhang et al., 2016; Tsutiya et al., 2017). In fact, abnormal NMDA receptor composition, including GluN2B and GluN1, is observed in hippocampus of *Dpysl2* knock-out (KO) mice resulting in a reduction of long-term potentiation (LTP) induction and in defects in learning function (Zhang et al., 2016). *Dpysl2^−/−^* mice also showed altered expression of proteins involved in GABAergic synapse (NSF, PRKACB, GNAI1), glutamatergic synapse (GRIA2, PRKACB, GNAI1, SHANK3, SHANK2, GRIA1) and neurotrophin signaling pathways (Table 2). These alterations of both inhibitory and excitatory synapse related proteins may contribute to the behavioral phenoytpe of these mice (Nakamura et al., 2016).

An altered LTP is found in CA1 hippocampi neurons of *Dpysl1^−/−^* and *Dpysl4^−/−^* mice models (Table 2; Su et al., 2007; Quach et al., 2008). Deletion of *Dpysl1* leads to a decrease in the expression of GAP43 and PSD95 proteins (Su et al., 2007) and inactivation of *Dpysl3* also disturbs the mRNA expression levels of genes encoding GluR1, GluR2, VgluT1, VgluT2, GABAɑ1, GABAAγ2, GABAB receptor 1 and vGAT, in a region-dependent manner (Tsutiya et al., 2017). To date, no reports have shown that DPYSL5 is required for LTP formation, but in cerebellum of *Dpysl5^−/−^* mice the induction of long-term depression (LTD) is deficient between parallel fibers and Purkinje cells (Table 2; Yamashita et al., 2011). Consequently, involvement of DPYSL in LTP and in LTD is a critical mechanism for memory and learning processes (Malenka and Bear, 2004; Stacho and Manahan-Vaughan, 2022).

**Figure 3:**
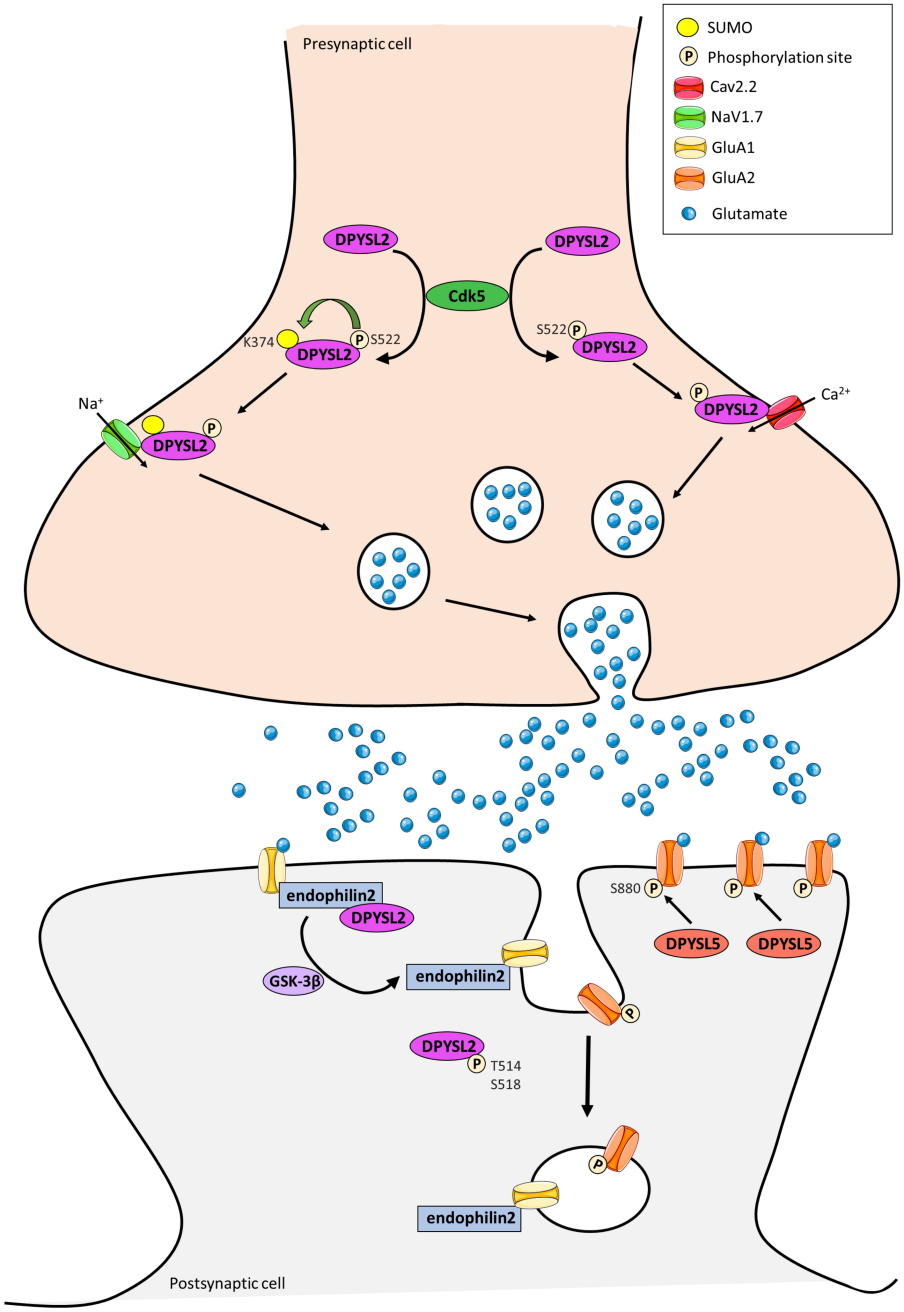
Representation of the contribution of DPYSL2 and DPYSL5 proteins in the control of synaptic plasticity. DPYSL2 binds and regulates the trafficking of both voltage gated Na^2+^ (NaV1.7) and Ca^2+^ (CaV2.2) channels at presynaptic terminal. DPYSL2 phosphorylation at Ser 522 by Cdk5 promotes its binding to Cav2.2. This interaction causes an increased number of CaV2.2 at cell surface leading to an increase in Ca^2+^ influx and glutamate release. SUMOylation is enhanced by phosphorylation of DPYSL2 through CDK5 action. This SUMOylation induces an increase of NaV1.7 channel at surface and in neuronal excitability. At postsynaptic level, DPYSL2 phosphorylation by GSK3β inhibits interaction with endophilin2 and reduces the number of GluA1 subunits of AMPARs at membrane. Similarly, DPYSL5 *via* GluA2 S880 phosphorylation can modulate traffic at the surface of the GluA2 subunit of AMPA receptors. (Adapted from Lin et al., 2019; Stratton et al., 2020; Henley et al., 2021).

In parallel, three studies showed the involvement of DPYSL proteins in the dynamic trafficking of AMPA receptors (AMPARs) (Khazaei et al., 2014; Lin et al., 2019) and NMDA receptors (NMDARs) (Bretin et al., 2006). It is well-known that the trafficking of glutamatergic receptor, which enables the endocytosis, recycling and exocytosis of receptors is crucial for synaptic strength and plasticity. Moreover, the interaction between dephosphorylated DPYSL2 and endophilin2 promotes insertion of the GluA1 subunit of AMPARs to the post-synaptic membrane and increases amplitude and frequency of mEPSCs in cultured hippocampal neurons (Figure 3; Zhang et al., 2018). In contrast, DPYSL2 downregulates the amount of the NR2B subunit of the NMDARs on the surface of cortical neurons (Bretin et al., 2006). DPYSL5 protein also regulate the endocytosis of GluA1 subunit of the AMPARs *via* phosphorylation of GluA2 at Serine 880, illustrating a specific function of DPYSL5 at glutamatergic synapses (Lin et al., 2019; Figure 3). On the other hand, it is also shown that DPYSL5 could modulate GluA2 endocytosis *via* GluA2 phosphorylation site on Serine 880 (S880), triggering social deficit (Lin et al., 2019).

Complementary to their role at the postsynaptic level, DPYSL are also expressed at presynaptic terminal. DPYSL2 and DPYSL4 have been identified as main regulators of ion currents voltage dependent (Brittain et al., 2009; Quach et al., 2011, 2013). Alike, DPYSL4 facilitates the depolarization-evoked Ca^2+^ response of L- and N-type Ca^2+^ channels to promote dendrite morphogenesis of hippocampal neurons (Quach et al., 2013; Figure 3). DPYSL2 binds and regulates the trafficking to membrane of both presynaptic voltage-gated Na^2+^channels (NaV1.7) (Dustrude et al., 2013, 2016, 2017) and N-type voltage-gated Ca^2+^ channel (CaV2.2) (Brittain et al., 2009, 2011; Moutal et al., 2016).

Several post-translational modifications of DPYSL2 allow modulation of membrane addressing. For instance, DPYSL2 phosphorylation at Serine 522 by Cdk5 promotes association between DPYSL2 and cytoplasmic loops of CaV2.2 (Brittain et al., 2012; Chew and Khanna, 2018), leading to an increase of Ca^2+^ influx through the Cav2.2 channel and the release of neurotransmitters (Brittain et al., 2009, 2012). Similarly, SUMOylation of DPYSL2 alters calcium influx (Ju et al., 2013) and increases cell surface expression of NaV1.7 channel (Dustrude et al., 2016). These findings suggest that DPYSL2 can regulate synaptic activity and plasticity by modifying the membrane localization of ion channels and thus controlling associated currents (Figure 3). Moutal et al. identified syntaxin1 as a novel DPYSL2 protein partner (Moutal et al., 2017), and this protein is involved in synaptic vesicle endocytosis neurotransmitter release (Rizo, 2022).

Interestingly, DPYSL3 interacts with proteins involved in synaptic vesicle recycling (Quinn et al., 2003) and electrophysiological experiments demonstrated that DPYSL3 enhances Ca^2+^ current density in hippocampal neurons (Wang et al., 2010).

Together, the DPYSL proteins act as neuromodulators of Ca^2+^ channel function and seem to play a major role in synaptic vesicle exocytosis and transmitter releasing in synaptic cleft (Figure 3).

Thus, the combination of these findings converged on the fact that DPYSL proteins might be key regulators of synapses architecture and activity *via* an interaction with cytoskeletal proteins but also with synaptic scaffolding proteins. Future protein interaction studies shall further clarify DPYSL protein interactome at the synapses.

## 7. *DPYSL* genes and neurodevelopmental disorders

Consistently with the major role of DPYSL proteins in dendritic organization and in formation and maturation of synapse, various studies suggested that they would contribute in the pathophysiology of psychiatric diseases such as schizophrenia and NDDs (Table 3; Edgar et al., 2000; Charrier et al., 2003; Hong et al., 2005; Beasley et al., 2006; Bader et al., 2012; Braunschweig et al., 2013; Yamashita et al., 2013; Lee et al., 2015; Quach et al., 2015; Tsutiya et al., 2017; Quach et al., 2021; Murtaza et al., 2022) (database SFARI, denovo-db). Interestingly, dendritic and spine dysfunctions are described in NDDs including schizophrenia, Down’s syndrome, Fragile X syndrome, Rett syndrome and ASD (Huttenlocher, 1991; Kaufmann and Moser, 2000; Martínez-Cerdeño, 2017; Nelson and Bender, 2021; Quach et al., 2021). *Dpysl* KO mouse models displayed morphological abnormalities in neurons as well as behavioral defects similar to those found in schizophrenia (hyperactivity, learning and memory deficits...) or in ASD (Yamashita et al., 2013; Nakamura et al., 2016; Tsutiya et al., 2017; Ohtani-Kaneko, 2019).

**Table 3:** Summary table of *de novo* heterozygous missense variants in *DPYSL* genes and their contribution in neurodevelopmental diseases.

Genes	Allele change	Residue change	Behavioral phenotype	References
*DPYSL1*	c.1052T > C	p.Phe351Ser	ID, behavioral problems	Ravindran et al. (2022)
	c.1280C > T	p.Thr427Met	ASD, no ID, delayed motor development	Ravindran et al. (2022)
	c.1766C > T	p.Pro589Leu	ID, ASD, delayed motor development	Ravindran et al. (2022)
*DPYSL2*	c.42C > A	p.Ser14Arg	ID	Suzuki et al. (2022)
	c.1028G > A	p.Arg343His	ASD	Satterstrom et al. (2020); Database: SFARI
	c.1312C > A	p.His438Asn	ASD	De Rubeis et al. (2014); Database: SFARI, *de novo*-db
	c.1693C > T	p.Arg565Cys	ID	Suzuki et al. (2022)
	c.1801C > T	p.Arg601Cys	ASD	Iossifov et al. (2014); Database: SFARI, *de novo*-db
*DPYSL3*	c.415G > A	p.Val139Ile	ASD	Iossifov et al. (2014); Database: SFARI, *de novo*-db
	c.1622C > A	p.Ser541Tyr	ASD	Tsutiya et al. (2017)
*DPYSL5*	c.121G > A	p.Glu41Lys	Severe ID Behavioral problems	Jeanne et al. (2021); Database: *de novo*-db
	c.139G > A	p.Gly47Arg	ID Ritscher-Schinzel syndrome	Jeanne et al. (2021)
	c.241G > A	p.Asp81Asn	Developmental disorder	Database: *de novo*-db
	c.1090G > A	p.Val364Ile	ASD	Database: *de novo*-db

Fragile X mental retardation protein (FRMP) encoded by the *FMR1* gene, is an RNA binding protein involved in fragile X syndrome, and regulates the function of many neuronal mRNAs crucial for neuronal development, synaptic plasticity and dendritic spine architecture (Banerjee et al., 2018). Interestingly, a proteomic analysis on extracts of nucleus laminaris from chicken identified CRMP1 and DPYSL2 as candidate substrates for FMRP (Sakano et al., 2017). Post-transcriptional modifications of DPYSL proteins, such as SUMOylation, impact their function in synapse, which is of particular interest for Fragile X syndrome since the activation of mGluR5 receptors promotes the SUMOylation of FRMP, leads to the dissociation of FRMP from mRNA granules to regulate spine elimination and maturation (Khayachi et al., 2018). Moreover, DPYSL2 protein expression can be controlled by the mTOR signaling pathway that is dysregulated in fragile X syndrome (Sharma et al., 2010). Both DPYSL2 and mTOR are associated with common physiological functions such as neuronal polarity, axonal outgrowth and synaptic strength as well as brain disorders including schizophrenia (Pham et al., 2016; Na et al., 2017; Izumi et al., 2022). Taken together, these data suggest that deregulation of the mTor-DPYSL2 molecular pathway may be involved in NDDs such as schizophrenia or ID.

For instance, genetic variants of *DPYSL1* or *DPYSL2* genes and alteration of DPYSL1 and DPYSL2 proteins levels have been reported in post-mortem brains of schizophrenic patients (Beasley et al., 2006; Martin-de-souza et al., 2010; Nomoto et al., 2021). Additionally, a link between DPYSL1 and DPYSL2 and the maternal antibody-related ASD subtype (MAR ASD) has been established (Braunschweig et al., 2013; Ramirez-Celis et al., 2021). Maternal antibodies in the placenta target fetal proteins and would cause alterations in neurodevelopment leading to behaviors associated with autism. A recent study highlighted that maternal IgG reactivity during pregnancy to both DPYSL1 + DPYSL2 increased at 16-fold the odds of an ASD diagnosis compared to the control group and over 6-fold relative to the ID group (Ramirez-Celis et al., 2022). This pattern DPYSL1 + DPYSL2 of MAR ASD is associated with ASD + ID diagnosis and ASD no-ID (Ramirez-Celis et al., 2022).

**Figure 4:**
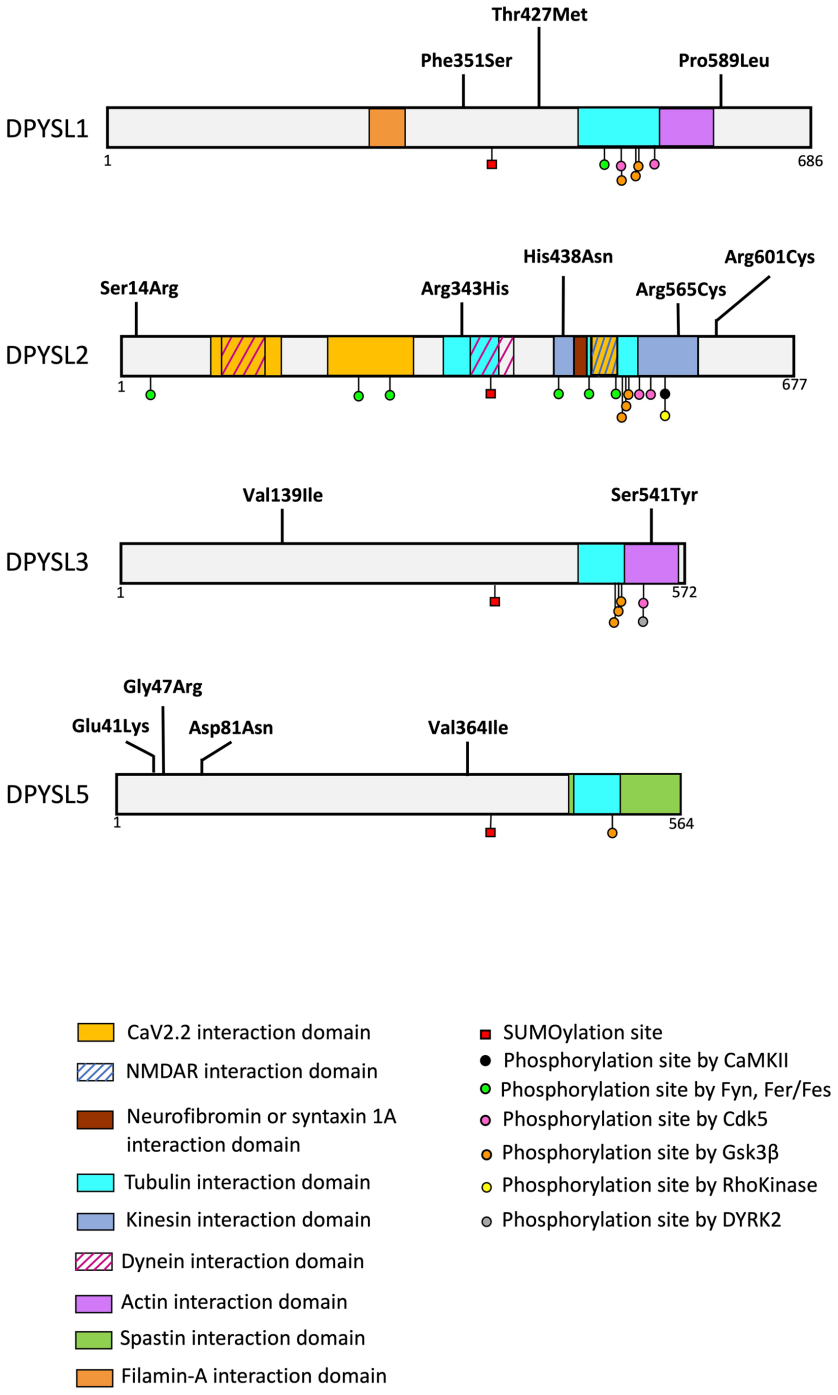
Schematic representation of protein interaction domains, post-translational. The interaction between DPYSL proteins and the represented proteins was identified in animal models (mouse, rat). Alignment of the amino acid sequences corresponding to these domains demonstrates sequence conservation in humans. The list of binding sites and post translational modifications provided in the figure is non-exhaustive. (Adapted from Nakamura et al., 2020).

In addition to DPYSL1-2, DPYSL3, and DPYSL5 are also involved in psychiatric disorders with the description of *de novo* missense mutations in *DPYSL2, 3,* and *5* in individuals with NDDs (Table 3 and Figure 4).

### 7.1. Genetic variants in *DPYSL1*

A recent study reported heterozygous *de novo* variants in the *DPYSL1* gene in three unrelated individuals with muscular hypotonia, ID and/or ASD (Table 3, Figure 4) (Ravindran et al., 2022). Whole exome sequencing identified two variants associated with ID (p.Pro589Leu for the long isoform of *DPYSL1* or p.Pro475Leu for the short isoform; p.Phe351Ser for the long isoform or p.Phe237Ser for the short isoform), and one variant in an individual with ASD (p.Thr427Met for the long isoform or p.Thr313Met for the short isoform). These variants are predicted to affect the ternary structure of *DPYSL1* and to impact the oligomerization of DPYSL1 proteins. When using the short isoform of *DPYLS1* protein, the p.Thr313Met and p.Pro475Leu variants are positioned next to the dimer/tetramer interface of CRMP1B, and they impair the homo-oligomerization of *DPYSL1*. The overexpression of variants p.Thr313Met and p.Pro475Leu in mouse cortical neurons caused a decrease in neuritic outgrowth (Ravindran et al., 2022), which is a morphological phenotype associated with many neurodevelopmental disorders (Quach et al., 2015; Prem et al., 2020). Interestingly, *Dpysl1^−/−^* mice have defects in dendritic spines (Yamashita et al., 2007; Makihara et al., 2016) and an inability to induce LTP (Su et al., 2007). In addition, these mice show schizophrenia like behaviors (Yamashita et al., 2013).

### 7.2. Genetic variants in *DPYSL2*

Three *de novo* missense variants in *DPYSL2*, predicted deleterious *in silico*, were described in individuals with ASD from the Autism Sequencing consortium (variant p.His438Asn) (Veron et al., 2018) and in two individuals of the Simons Simplex Collection (p.Arg343His, p.Arg601Cys) (Iossifov et al., 2014; Satterstrom et al., 2020). Of interest, a recent study described two unrelated patients with ID and hypoplasia of the corpus callosum associated *with a de novo* missense variant (p.Ser14Arg or p.Arg565Cys) of *DPYSL2* (Table 3 and Figure 4; Suzuki et al., 2022).

Functional assays in zebrafish model showed that p.Ser15Arg and p.Arg566Cys variants (corresponding to codons Ser14 and Arg 565 of human *DPYSL2*) led to the loss of function of DPYSL2 protein. Cell transfection experiments of DPYSL2 protein variants demonstrated that both mutations caused a decrease in DPYSL2 protein levels, probably due to increased degradation by the proteasome. Moreover, both variants impaired DPYSL2 interaction with tubulin. These results collectively support the pathogenity impact of p.Ser14Arg and p.Arg565Cys variants causing intellectual disability in humans (Suzuki et al., 2022). It is interesting to note that the patients described by Suzuki et al., have dysplasia of the corpus callosum which has also been found in *Dpysl2^−/−^* mouse model that display a dysgenesis of corpus callosum and defects in callosal axon guidance (Ziak et al., 2020).

In mice, *Dpysl2* deficiency induces a reduction of spine density and dendritic branching in CA1 hippocampal neurons and in layer V of cortical neurons of mice (Makihara et al., 2016; Zhang et al., 2016). Moreover, brain-specific *Dpysl2-*KO mice display hyperactivity and social, cognitive and affective behavioral impairments, reminiscent of deficits associated with schizophrenia (Zhang et al., 2016). On the other hand, total deletion of *Dpysl2* in mice leads to histological and behavioral alterations similarly to “ASD-related phenotype” such as axonal pruning defects and inadequate elimination of dendritic spines in dentate gyrus of hippocampi (Ziak et al., 2020). Very interestingly, a defect in synaptic pruning in layer V pyramidal neurons has been reported in temporal lobe of postmortem ASD patients (Tang et al., 2014). Furthermore, *Dpysl2^−/−^* mice exhibit ASD-related social behavior changes such as ultrasonic vocalization deficits in the early postnatal period (P8, P12) and social behavioral deficits in adult (Ziak et al., 2020).

### 7.3. Genetic variants in *DPYSL3*

The genetic analysis of the Simon Simplex Collection reported two *de novo* missense variants (p.Ser541Tyr and p.Val139Ile) of the *DPYSL3* gene associated with ASD (Iossifov et al., 2014; Tsutiya et al., 2017; Table 3 and Figure 4).

The study of *Dpysl3-*KO cultured hippocampal neurons showed that *DPYSL3* deficiency was associated with longer dendrites with more branching (Tsutiya et al., 2017). The *Dpysl3*-KO neurons transfected with pEGFP-DPYSL3^S540Y^ exhibited an increasing in dendritic branching compared to control *Dpysl3*-KO neurons transfected with pEGFP-DPYSL3^WT^ (the human DPYSL3 Serine 541 corresponds to mouse DPYSL3 codon Serine 540). To conclude, the p.Ser541Tyr mutation alters dendritic morphology and impairs the function of DPYSL3 (Tsutiya et al., 2017).

In addition, *Dpysl3-*KO mice exhibit several ASD-like phenotypes, including deficits in social interaction (determined by the three-chambers test) and alterations of sensory response measured by the emission of ultrasonic vocalization of mouse pups after different sensory stimuli. Interestingly, the serine 541, which is mutated into Tyrosine in an ASD patient (Tsutiya et al., 2017), is a phosphorylation site of DPYSL3 (Figure 4; Mertins et al., 2016) (database PhosphositePlus). As phosphorylation is essential for DPYSL cellular functions, it is plausible that DPYSL3 p.Ser541Tyr mutation may cause a loss-of-function of DPYSL3 leading to defects in dendritic arborization associated with behavioral deficits. Furthermore, Tsutiya and colleagues highlighted that *Dpysl3* deficiency altered mRNA expression of *Gria1* and *Gria2* (encoding GLUR1 and GLUR2 subunits of the AMPA receptor), essential for dendritic development and maturation (Chen, 2009). In addition, it remains essential to highlight that previous studies also revealed contribution of these two AMPA receptors subunits in mice with social deficits and in patients with ASD or other NDDs (Purcell et al., 2001; Ramanathan et al., 2004; Essa et al., 2013; Erickson et al., 2014; Uzunova et al., 2014; Kim et al., 2019).

### 7.4. Genetic variants in *DPYSL5*

The *DPYSL5* gene (and its respective protein DPYSL5) is the latest discovered member of DPYSL family (Fukada et al., 2000; Inatome et al., 2000; Ricard et al., 2001), and has been recently described as a novel candidate gene for NDDs (Table 3 and Figure 4). An international collaboration allowed to identify nine families including patients with ID associated with cerebral malformations, and carriers of *de novo* heterozygous missense variants in *DPYSL5.* A recurrent *de novo* variant p.Glu41Lys has been identified in eight unrelated subjects with ID, corpus callosum agenesis and posterior fossa abnormalities. Furthermore, a p.Gly47Arg variant was found in two sisters with Ritscher-Schinzel syndrome (Jeanne et al., 2021). It is critical to note that all individuals with p.Glu41Lys and p.Gly47Arg mutations in *DPYSL5* display an agenesis of corpus callosum which is a neuroanatomical malformation already associated with ASD and ID (Halgren et al., 2012; Wegiel et al., 2018; Li et al., 2019; Mimura et al., 2019; Nabais Sá et al., 2020; Qi et al., 2022).

Very interestingly, a dysgenesis of the corpus callosum has also been described for the two patients with ID and carrying the mutations p.Ser14Arg and p.Arg565Cys in *DPYSL2* gene. Another similarity worth mentioning is a hypoplasia of the cerebellum in patients carrying both variants of *DPYSL5* which is also found in the patient with mutation p.Ser14Arg of *DPYSL2* gene (Jeanne et al., 2021; Suzuki et al., 2022). DPYSL5 protein may form a homo- or hetero-tetramer with DPYSL2-4 (Wang and Strittmatter, 1997; Stenmark et al., 2007). As previously described in Jeanne et al. publication, *DPYSL5* p.Glu41Lys and p.Gly47Arg variants do not affect oligomeric assembly but by adding a positive charge to the electrostatic surface of the protein, which may alter the interaction between DPYSL5 and its partners (Jeanne et al., 2021). It is well-characterized that primary neuronal cultures, overexpressing DPYSL5 inhibits tubulin polymerization and neurite growth (Brot et al., 2014). However, overexpression of missense variants of DPYSL5 results in the loss of the inhibitory regulation of DPYSL5 on dendritic growth. Both mutations altered the function of DPYSL5 by preventing the formation of the complex with MAP2 and βIII-Tubulin. In addition, p.Gly47Arg substitution increased the binding of DPYSL5 to DPYSL2 (Jeanne et al., 2021). Thus, it has been hypothesized that p.Gly47Arg modulates the neuronal function of DPYSL2 by increasing the formation of DPYSL2/DPYSL5 complex. This study highlighted the importance of DPYSL5 in neuronal development and put forward that defect in these regulatory mechanisms is responsible for a syndromic form of NDD with brain anomalies.

No studies have reported behavioral defects related to NDDs in *Dpysl5* mutant animal models but Lin et al., demonstrated that the hippocampal overexpression of DPYSL5 triggers social interaction deficits in both control mice and in 3xTg-Ad mice, a classical mouse model of Alzheimer’s disease (Lin et al., 2019) suggesting that DPYSL5 closely controls social behavior. Overall results reveal that impairments in DPYSL2, DPYSL3 and DPYSL5 functions can lead to ID, ASD or schizophrenia. Although genetic causes of ASD and ID include mutations in genes coding for proteins involved in various pathways, such as chromatin remodeling, transcriptional regulation or the dynamics and reorganization of the cytoskeleton, a majority of genes/proteins mutated in NDDs contribute to the architecture and activity of the synapses (Guilmatre et al., 2009; Pavlowsky et al., 2012). Thus, in this review we have provided compelling evidence that dysregulation of DPYSL expression may also impair synaptic function and consequently lead to early-onset cognitive disorders, demonstrating that DPYS*L* genes and proteins defects may also contribute to “synaptopathies.”

## 8. Discussion

The DPYSL proteins family appear to be involved in various biological events during the development including differentiation, axon guidance, neurites extension, dendritic branching and axonal regeneration (Ip et al., 2014). Here, we gathered various evidence from an extensive review of the literature that DPYSL genes and proteins are necessary for regulating the formation and the maturation of synapses, the neurotransmission and synaptic plasticity, mainly due to their synaptic localization at both pre and post synaptic terminals (Collins et al., 2006; Laumonnier et al., 2007; Brittain et al., 2009, 2011).

Genetic deletion of *Dpysl* in mice leads to synaptic impairment as well as cognitive and behavioral disorders, which are common defects associated with NDDs. As summarized in Table 3, genetic studies uncovered the contribution of *de novo* missense mutations in the *DPYSL* genes in NDDs (Iossifov et al., 2014; Veron et al., 2018; Satterstrom et al., 2020; Jeanne et al., 2021; Suzuki et al., 2022) (database: SFARI; *denovo*-db), suggesting their central role in the brain formation and functioning and the pathogenesis of NDDs. This review also highlights that DPYSL may have antagonistic or complementary activity and that their predisposition to homo- and hetero-oligomerization may have a direct impact on their physiological role. It is likely that the localization of variants in specific interaction and/or functional domains necessary for oligomerization of DPYSL proteins may have a consequence on their synaptic functions and thus lead to NDD.

Further understanding of signaling pathways located upstream and downstream of DPYSL for each homo or hetero-tetramer assembly will likely help to elucidate the physiological contribution of DPYSL proteins during brain formation and maturation and the pathogenic mechanisms leading to neurodevelopmental disorders.

## Online database

BBI-Allen single cell atlases, https://descartes.brotmanbaty.org/ (accessed January 19, 2023).

National Center for Biotechnology Information, https://www.ncbi.nlm.nih.gov/ (accessed January 19, 2023).

SFARI gene, https://gene.sfari.org/ (accessed January 19, 2023).

Denovo-DB, https://denovo-db.gs.washington.edu/denovo-db/ (accessed January 19, 2023).

PhosphoSitePlus, https://www.phosphosite.org/ (accessed January 19, 2023).

## Author contributions

FD and FL wrote the first draft of the manuscript. DU, PV, and MJ contributed in the revision of the initial version. All authors revised and approved the final version of the manuscript submitted for publication.

## Funding

FD was supported by a PhD fellowship from the Région Centre Val de Loire. Work in the lab of FL devoted to DPYSL is supported by the Inserm (GOLD cross-cutting program on genomic variability), the Association pour le Développement de la Neurogénétique.

## Conflict of interest

The authors declare that the research was conducted in the absence of any commercial or financial relationships that could be construed as a potential conflict of interest.

## Publisher’s note

All claims expressed in this article are solely those of the authors and do not necessarily represent those of their affiliated organizations, or those of the publisher, the editors and the reviewers. Any product that may be evaluated in this article, or claim that may be made by its manufacturer, is not guaranteed or endorsed by the publisher.
